# The effectiveness of rebamipide mouthwash therapy for radiotherapy and chemoradiotherapy-induced oral mucositis in patients with head and neck cancer: a systematic review and meta-analysis

**DOI:** 10.1186/s40780-019-0146-2

**Published:** 2019-07-25

**Authors:** Shinsuke Akagi, Takashi Fujiwara, Mai Nishida, Akiko Okuda, Yuka Nagao, Toshikatsu Okuda, Hidenori Tokuda, Kazunobu Takayanagi

**Affiliations:** 1 Department of Pharmacy, Ohara Healthcare Foundation Kurashiki Central Hospital, Okayama, Japan; 2 Department of Otolaryngology/Head and Neck Surgery, Ohara Healthcare Foundation Kurashiki Central Hospital, Okayama, Japan

**Keywords:** Meta-analysis, Rebamipide, Mouthwash, Mucositis, Chemoradiotherapy, Chemotherapy, Adverse event, Head and neck cancer

## Abstract

**Background:**

Oral mucositis is a frequent and severe adverse event in patients undergoing chemoradiotherapy for head and neck cancers, especially grade 3 or 4 mucositis. Occurrence may result in drop-out from treatment, thereby reducing survival. We aimed to clarify the effectiveness and safety of rebamipide mouthwash for oral mucositis in patients with head and neck cancer receiving treatment.

**Methods:**

We carried out a systematic review and meta-analysis of patients with head and neck cancer who were treated with rebamipide mouthwash. We searched PubMed, EMBASE, and Cochrane Central Register of Controlled Trials (CENTRAL), and the World Health Organization (WHO) International Clinical Trial Registry Platform. The primary outcome was the incidence of severe oral mucositis, and secondary outcomes were time from treatment start to onset of oral mucositis, the response rate of radiotherapy, and any adverse events.

**Results:**

We included three studies comparing rebamipide versus placebo, all of which evaluating chemoradiotherapy induced oral mucositis. The chemotherapeutic agent was docetaxel in one study and cisplatin in the remaining two. Radiotherapy in each study consisted of 3D-conformal radiation therapy, intensity modulated radiation therapy and conventional radiation therapy, respectively. The calculated odds ratio was 0.29 [95% confidence interval (CI): 0.15 to 0.55], showing a positive association in the three studies between the incidence of grade 3–4 oral mucositis and chemotherapy for head and neck cancer. One study reported an onset of oral mucositis and the time to onset was 14.6 ± 6.4 days for the rebamipide group and 11.2 ± 4.4 days for placebo. One study reported a complete response of 8.3% for placebo and 16.7% for the rebamipide the group, and the partial response was 91.7 and 75.0%, respectively. Adverse events were reported in two studies to be 6.1 and 11.6% for placebo, and 19.4 and 26.0% in the rebamipide group, respectively.

**Conclusions:**

Rebamipide mouthwash is effective in the prevention of severe mucositis and stomatitis. However, evaluation of adverse events in observational studies are needed.

## Introduction

Radiotherapy and surgery are the most effective treatments in head and neck cancer. Radiotherapy and surgery have a similar cure rate in early-stage cancer, but radiotherapy is better able to preserve organ function, with combined surgery and radiotherapy improving the prognosis in cases of advanced cancer [1]. The role of radiotherapy has broadened, as a result of developments in computer software, radiation delivery technology, and combined chemotherapy.

Oral mucositis is a severe and frequent adverse event of chemoradiotherapy for head and neck cancer, with 30–50% of patients experiencing grade 3 or 4 mucositis during treatment [2–4]. Oral mucositis results in an impaired mucosal barrier, and is associated with a longer hospitalization duration due to infection [5]. Oral mucositis is effectively treated with frequent oral rising, and several medical therapies are widely used, including mucosal coating agents, anti-inflammatory agents and topical granulocyte macrophage colony stimulating factor [5]. However, oral mucositis is still remains a critical and frequent issue for those undergoing head and neck cancer with chemoradiotherapy.

Rebamipide is a mucosal protection drug, which is used for gastiritis and gastric ulcer in a number of Asian countries [6]. Matsuda et al. first reported the efficacy of rebamipide for oral mucositis in 1994, and the subsequent rebamipide mouthwash, developed by the same authors [7], has been used for mucositis caused by Behcet’s disease [5], chemotherapy [8], and radiotherapy [9]. A pilot randomized controlled trial (RCT) in 2012 reported that rebamipide mouthwash reduced severe oral mucositis induced by radiotherapy or chemoradiotherapy [9]. Since then, there has been no further validation studies, but new trials have been published in 2017 [10, 11]. A Cochrane systematic review of oral mucositis for patients with cancer was published in 2011 [12], but the review did not include rebamipide mouthwash and has not yet been updated. Because there is a lack of a relevant systematic review on this topic, the aim of this review was to assess the effectiveness of rebamipide mouthwash in patients with oral mucositis receiving radiotherapy or chemoradiotherapy.

## Methods

We carried out a systematic review and meta-analysis of patients with head and neck cancer who were treated with rebamipide. Standard guidelines for systematic review were used [13]. The protocol of this review was registered with International Prospective Register of Systematic Reviews (PROSPERO: http://www.crd.york.ac.uk/PROSPERO/) under registration number No 76566.

### Eligibility criteria

We included all adult and child patients diagnosed with head and neck cancer, who were treated with radiotherapy or chemoradiotherapy and underwent therapy with rebamipide gargle, rinse or spray. Patients with both primary and recurrent head and neck cancer were included. Radiotherapy or chemoradiotherapy included preoperative, postoperative, and sole. We included RCTs and cluster-RCTs. We excluded cluster-RCTs which included only two clusters, crossover trials, and quasi-RCTs.

### Search strategy

We searched the Pubmed, EMBASE, and Cochrane Central Register of Controlled Trials CENTRAL) (up to November 2018), and World Health Organization (WHO) International Clinical Trial Registry Platform databases. Medical subject headings and text words as terms in the searches were “rebamipide”, “head and neck neoplasm”, “otorhinolaryngologic neoplasms”, “radiotherapy” and “randomized control trial”.. Both published and unpublished studies in all languages prior to November 2018 were included. The search strategies and search results of each database were registered at PROSPERO.

### Outcomes

The primary outcome was incidence of severe oral mucositis defined as either grade 3–4 of WHO oral toxicity scale [14], Radiation Therapy Oncology Group (RTOG) scale [15], or National Cancer Institute Common Toxicity Criteria (NCI-CTC) scale.

The secondary outcomes were time from treatment start to onset of oral mucositis, response rate (complete and partial response) of radiotherapy defined by the Response Evaluation Criteria in Solid Tumors (RECIST) [16], and any adverse events defined in each article by the authors were collected.

### Study selection and data extraction

Three individual authors (SA, TF and MN) reviewed all titles and abstracts identified by electronic searches. We obtained the full text of studies that potentially met the eligibility criteria. Three authors independently assessed the eligibility of the studies from the full text. Any disagreements were resolved by discussion. We consulted another author if disagreement was not resolved by discussion. Three authors also extracted the following characteristics from the studies: patients including population (age, sex); primary site of cancer; Tumor Nodes Metastasis (TNM) classification of cancer; primary or recurrent cancer; type of radiotherapy (definitive, adjuvant, pre-operative, or postoperative); radiation technique [conventional, 3 dimensional conformal radiation therapy (3D-CRT), or intensity modulated radiation therapy (IMRT); regimens of rebamipide mouthwash.

### Study quality assessment / risk of bias across studies

SA, TF and MN independently assessed the risk of bias of the included studies in following items: sequence generation, allocation concealment, blinding of participants and personnel, blinding of outcome assessment, incomplete outcome data, selective outcome reporting, and other sources of bias. We used the Cochrane ‘Risk of bias’ tool, which involved describing each of these domains as reported in the trial and then assigning a judgement about the adequacy of each entry as ‘low’, ‘high’ or ‘unclear’ risk of bias.

### Data analysis

We performed a statistical analysis of the outcomes using RevMan 5.3. We calculated odds ratios (OR) and 95% confidence interval (CI) for dichotomous outcomes, and calculated mean difference and 95% CI for continuous outcomes. For adverse eventts, we did not conduct meta-analysis and state narratively. For missing or unavailable data, we contacted the study authors. For data synthesis, we used available case analyses. A random-effect model was selected to perform statistical analyses within and between the heterogeneity of studies [17]. Chi-square test and *I*^2^ statistical analysis were used to qualitatively describe the heterogeneity and quantitatively estimate the proportion of the overall variation, respectively [17]. We conducted pre-specified subgroup analysis in primary outcome. We planned subgroup analysis of concentration of rebamipide (comparing different concentration of rebamipide) and type of radiotherapy (radiotherapy versus chemoradiotherapy), but we could not conduct subgroup analysis of type of radiotherapy because all included studies enrolled chemoradiotherapy patients. When included studies had more than two intervention groups (e.g. different concentrations of rebamipide), we split the control group into two or more groups with smaller sample sizes to make a reasonably independent comparisons.

## Results

### Study selection

**Fig. 1:**
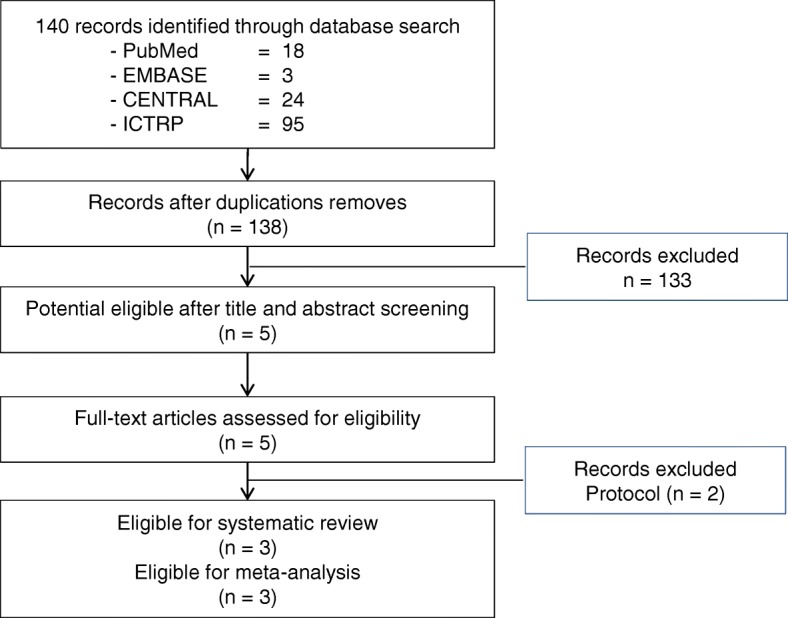
Process of study selection

Of the 140 potential citations, 5 articles were eligible for a full-text screening. After full-text screening, we identified three studies which met the criteria for eligibility. Figure 1 shows the process of study selection.

### Details of included studies

**Fig. 2:**
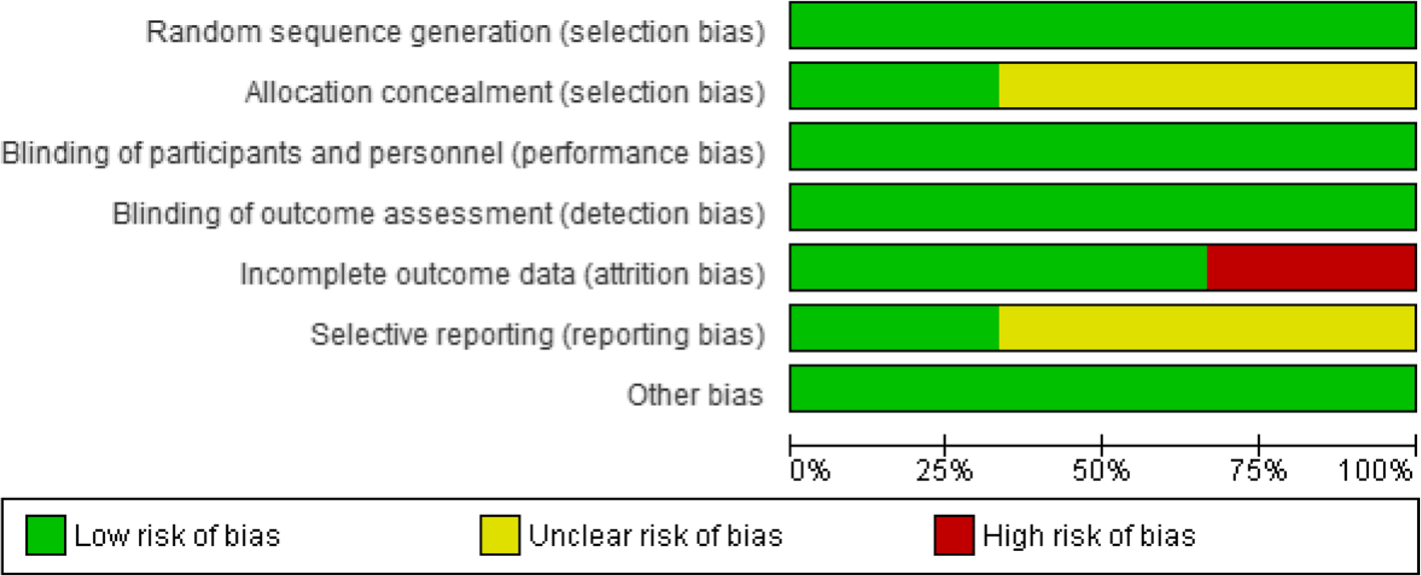
Risk of bias graph: review authors’ judgement about each risk of bias item presented as percentages across all included trials

Table 1 shows the characteristics of the included studies. Three studies compared rebamipide gargle versus placebo. Two studies used the same gargle solution [9, 11], and one study used liquid [10]. All studies evaluated chemoradiotherapy-induced oral mucositis. One study used docetaxel and two studies used cisplatin. Two studies recruited participants from 2014 who underwent 3D-CRT or IMRT [10, 11]. One study recruited participants from 2005 and conventional radiotherapy was used [9], and conventional radiotherapy was undertaken preoperatively, despite this being recently uncommon [1]. Oral mucositis was assessed 4 weeks after chemoradiotherapy in one study and at the end of chemoradiotherapy in two studies. Overall, the methodological qualities of included studies were adequate, although concealment methods were unclear in two studies. One study had a 34% dropout, and the reasons included the patient’s request (*n* = 23), adverse events (*n* = 5), and physician’s judgment (*n* = 4) [10]. Risk of bias results are shown in Fig. 2.

**Table 1:** Characteristics of included studies

	Yasuda 2011	Chaitanya 2017	Yokota 2017
Characteristics of patients			
Number of patients	24	60	94
Mean age (years)	60.4	51.5	61.0
Dropout	0 (0.0%)	4 (6.7%)	32 (34.0%)
Sex (Male/Female)	14/10	59/1	77/17
Site of cancer	Oral cavity	Head and neck squamous cell carcinoma	Head and neck cancer, primary tumor
TNM staging			
T1–2 Nany T3–4 Nany	14 8^a^	unclear	58 36
Type of radiation	Definitive and preoperative	Definitive and post-operative	Definitive or post-operative
Combination with chemotherapy	Weekly docetaxel (10 mg/m^2^)	Weekly CDDP (40 mg/ m^2^) or Tri-weekly CDDP (100 mg/ m^2^)	Tri-weekly CDDP (80-100 mg/ m^2^)
Radiation technique	Conventional 2 Gy/fraction ≥40 Gy irradiation (total)	3D-CRT or IMRT 2 Gy/fraction in 6–7 weeks Total 60–70 Gy	3D-CRT or IMRT ≤2.2 Gy/fraction ≥60 Gy
Intervention of rebamipide			
Type of mouthwash	Gargle	Gargle	Liquid, rinse and swallow
Regimens of rebamipid	0.1% concentration 6 times daily	0.1% concentration 6 times daily	2 and 4% concentration 6 times daily
Control	Placebo	Placebo gargle	Placebo liquid
Timing of outcomes	At 4 weeks	At the end of chemoradiotherapy	At 57 days

Cisplatin (CDDP)

^a^TNM stages were unclear in 2 patients in Yasuda 2011

### Incidence of grade 3–4 oral mucositis

**Fig. 3:**
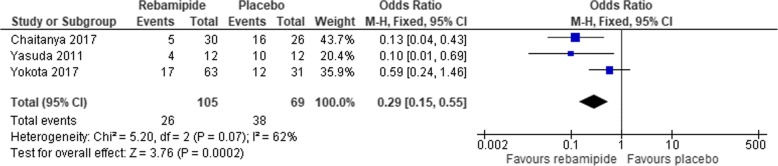
A forest plot of meta-analysis of a comparison of incidence of grade 3–4 mucositis between rebamipide and placebo

Three studies evaluated incidence of grade 3–4 oral mucositis and the pooled odds ratio was 0.29 (95% CI:0.15 to 0.55). Forrest plot of incidence of grade 3–4 oral mucositis was shown in Fig. 3.

### Time to onset of oral mucositis

One study evaluated onset of oral mucositis and the time to onset were 14.6 ± 6.4 days in rebamipide and 11.2 ± 4.4 days in placebo [11].

### Response rate of radiotherapy

One study evaluated adverse events using Medical Dictionary for Regulatory Activities (MedDRA) system organ class and preferred terms [11]. Total numbers of adverse events were 16.1% in placebo and 19.4% in rebamipide. One study reported complete and partial radiotherapy response rates in placebo (8.3 and 91.7%) and repamipide groups (16.7 and 75.0%), respectively [9].

### Any adverse events

Chaitanya et al. evaluated the pain intensity (range 1–10) was 4.2 ± 1.6 in rebamipide and 5.9 ± 2.2 in placebo. Yokota et al. evaluated any adverse events and reported that the incidence of adverse events potentially related to the study drug was 11.6% in placebo, 26% in 2% rebamipide and 13% in 4% rebamipide [10].

## Discussion

Rebamipide has been used clinically for the purpose on improving mucosal lesions such as gastric ulcer treatment, erosion during gastritis, hemorrhage, redness, and edema. Rebamipide is effective for oral complications caused by cancer and its treatment. Rebamipide increased the generation of gastric mucosal prostaglandin activity [18, 19], increased the volume of mucosal mucus by the synthesis of mucous polymeric glycoprotein not involved in prostaglandin [20], directly eliminated hydroxyl radicals, and suppressed of leukocyte superoxide production [21–24].

Head and neck cancer has a higher incidence of oral complications associated with treatment than in general cancers [2–4]. Therefore, oral complications are important complications related to continuation of treatment and a decrease in patient’s quality of life (QOL). It has been shown to be useful that radiation therapy and chemotherapy are used in combination with locally advanced squamous cell carcinoma of the head and neck [25]. The incidence of mucositis is higher in concurrent radiochemotherapy than radiotherapy [26]. Management of oral complications is important.

In this meta-analysis, it was shown that rebamipide mouthwash was statistically reduced incidence of grade 3–4 oral mucositis. There was no difference in time to onset of oral mucositis in concurrent radiochemotherapy and radiotherapy alone [11].

In previous studies, There was no difference in response rate of chemoradiotherapy and incidence of adverse event in rebamipide mouthwash group and placebo [9, 10]. This may have resulted from problems in the safety profile of rebamipide mouthwash. However, time to onset of oral mucositis, response rate of radiotherapy and adverese events is not pooled, those are only outcome in each one study.

In this meta-analysis, there was a difference of 0.1 to 4% in the concentration of rebamipide mouthwash, The rebamipide concentration which shows the elimination of hydroxyl radicals and the suppressive action of leukocyte superoxide production is 10 mM to 1 mM [21, 23, 27, 28]. Since, 0.1% is 2.695 mM, it is considered to be a sufficient concentration as a direct concentration in the oral cavity. One study reported that it was a direct effect than the effect that was absorbed after gargle and reached the oral mucosa via blood stream [10]. There was no difference in effect at rebamipide concentrations of 2 and 4%, It is also considered that there was no difference because it has sufficient local concentration [10].

There was no comparison with other drugs in this meta-analysis. Palifermin and others are used for oral complications induced by cancer and its treatment. The problem is that there is no comparison and study with these another agents. The evaluation scles of oral mucositis used in each study were not iniform, therefore there was a lack of cositency.

It is shown that it is effective for pain [11], and improvement of QOL of patients is expected [29]. Opioids are used for stomatitis and mucositis in radiotherapy, but they have not been compared and studied, and this is a future subject for investigation. It is considered that the agent is retained on the damaged mucosal surface and exerts a protective action by washing in the mouth of rebamipide.

This meta-analysis had also some limitations. The sample size of each trial was small. The total number of patients were 178. The studies included in this meta-analysis used different types of radiation techniques, and these are influenced by tumor stage and site of cancer. There were variability in the chemotherapy regimens with different dosing schedules and different anticancer drugs. This may have resulted in high heterogeneity. The evaluation scales of oral mucositis used in each study were not uniform, therefore there was a lack of incosistency.

## Conclusion

This meta-analysis shows that gargling treatment with rebamipide is superior to placebo for the development of mucositis and stomatitis due to chemoradiation, especially for severe cases of Grade 3 of higher. However, in order to confirm these trials, well-designed analyses are needed, and evaluation of adverse events in observational studies are also needed.

## Data Availability

The dataset used for this meta-analysis are available from the corresponding author on reasonable request.

## Abbreviations

3D-CRT: 3 dimensional conformal radiation therapy
CDDP: Cisplatin
CI: Confidence interval
Cochrane CENTRAL: Cochrane Central Register of Controlled Trials
IMRT: Intensity modulated radiation therapy
MedDRA: Medical Dictionary for Regulatory Activities
NCI-CTC: National Cancer Institute Common Toxicity Criteria
PROSPERO: International Prospective Register of Systematic Reviews
QOL: Quality of life
RCT: Randomized cotrolled trial
RECST: Response Evaluation Criteria in Solid Tumors
RTOG: Radiation Therapy Oncology Group
TNM: Tumor Nodes Metastasis
WHO: World Health Organization
